# Complete Taiwanese Macaque (*Macaca cyclopis*) Mitochondrial Genome: Reference-Assisted *de novo* Assembly with Multiple k-mer Strategy

**DOI:** 10.1371/journal.pone.0130673

**Published:** 2015-06-30

**Authors:** Yu-Feng Huang, Mohit Midha, Tzu-Han Chen, Yu-Tai Wang, David Glenn Smith, Kurtis Jai-Chyi Pei, Kuo Ping Chiu

**Affiliations:** 1 Genomics Research Center, Academia Sinica, Taipei, Taiwan; 2 Institute of Biochemistry and Molecular Biology, National Yang-Ming University, Taipei, Taiwan; 3 National Center for High-Performance Computing, Hsinchu, Taiwan; 4 Department of Anthropology, University of California Davis, Davis, CA, United States of America; 5 Institute of Wildlife Conservation, College of Veterinary Medicine, National Pingtung University of Science and Technology, Pingtung, Taiwan; 6 College of Life Science, National Taiwan University, Taipei, Taiwan; 7 Institute of Systems Biology and Bioinformatics, National Central University, Jhongli, Taiwan; Sichuan University, CHINA

## Abstract

The Taiwanese (Formosan) macaque (*Macaca cyclopis*) is the only nonhuman primate endemic to Taiwan. This primate species is valuable for evolutionary studies and as subjects in medical research. However, only partial fragments of the mitochondrial genome (mitogenome) of this primate species have been sequenced, not mentioning its nuclear genome. We employed next-generation sequencing to generate 2 x 90 bp paired-end reads, followed by reference-assisted *de novo* assembly with multiple k-mer strategy to characterize the *M*. *cyclopis* mitogenome. We compared the assembled mitogenome with that of other macaque species for phylogenetic analysis. Our results show that, the *M*. *cyclopis* mitogenome consists of 16,563 nucleotides encoding for 13 protein-coding genes, 2 ribosomal RNAs and 22 transfer RNAs. Phylogenetic analysis indicates that *M*. *cyclopis* is most closely related to *M*. *mulatta lasiota* (Chinese rhesus macaque), supporting the notion of Asia-continental origin of *M*. *cyclopis* proposed in previous studies based on partial mitochondrial sequences. Our work presents a novel approach for assembling a mitogenome that utilizes the capabilities of *de novo* genome assembly with assistance of a reference genome. The availability of the complete Taiwanese macaque mitogenome will facilitate the study of primate evolution and the characterization of genetic variations for the potential usage of this species as a non-human primate model for medical research.

## Introduction

Genome assembly has been an area of interest for research groups worldwide since the initiation of the Human Genome Project in 1990. Genome assembly is critically important in the sense that it provides genomic maps to show the locations of protein-coding genes, noncoding genes, regulatory elements, as well as sequence sometimes regarded as “junk DNA” for the future study of gene expression (e.g., transcriptome analysis) and regulation (e.g., miRNA expression, and mapping of transcription factor binding sites and epigenetic modifications).

The complete mitochondrial genomes (mitogenomes) of about one-third Asian macaques [1–3] have been sequenced and assembled up-to-date. These include the mitogenomes of the Indian rhesus macaque (*Macaca mulatta*) [4–6], Chinese rhesus macaque (*M*. *mulatta lasiota*) [7], longtail/crab-eating macaque (*M*. *fascicularis*) from Mauritius, Malaysia and Indochina [6, 8], Tibetan macaque (*M*. *thibetana*) [6, 9], Assam macaque (*M*. *assamensis*) [10], lion-tailed macaque (*M*. *silenus*) [6], stump-tailed macaque (*M*. *arctoides*) [6, 11], Tonkean macaque (*M*. *tonkeana*) [6] and Japanese macaque (*M*. *fuscata*) [12], leaving only the mitogenome of Taiwanese (Formosan) macaque (*M*. *cyclopis*) remain unsequenced. The mitogenome of Taiwanese macaque could be an important piece in solving the puzzle of evolution of the fascicularis group.

The Taiwanese macaque is the only non-human primate species endemic to Taiwan and they are commonly found in forest habitat in lowland to mid-elevation (up to approximately 2,000 meter above sea level). Together with the Indian rhesus macaque, Chinese rhesus macaque, crab-eating macaque, and Japanese macaque, it belongs to the fascicularis group of macaque species [2, 13–16]. However, this classification is based on phenotypic characteristics (e.g., the morphology of male sex organ) that may be discordant with phylogeny based on nuclear loci, such as short tandem repeats (STRs) and partial mitochondrial DNA (mtDNA) sequences. Mitogenome phylogenetics has emerged as a superior approach for the study of primate relationships and evolution [17], and the level of genetic differences among macaques justifies their usage as animal models for the study of different human diseases [18]. Therefore, completion of a whole mitogenome sequence for *M*. *cyclopis* is of immense interest and will facilitate the phylogenetic study of primate evolution and medical research.

Previous mitochondrial genome sequencing employed three types of approaches: capture-enrichment, next-generation sequencing (NGS) or a mixture of the two [19]. Initially, the most common approach used multiplex PCR to capture overlapping fragments that could be assembled into longer sequence [4, 7, 9, 10]. Later, amplification of longer fragments by long-range PCR or multiplex PCR [8] and NGS [20–22] was employed for assembly.

We constructed a *M*. *cyclopis* whole genome shotgun library by NGS. Taking advantage of the availability of *M*. *cyclopis* mitochondrial sequence reads in the library and using the existing *M*. *mulatta* mitogenome as a reference, we adopted a novel procedure of reference-assisted *de novo* assembly with multiple k-mer strategy to complete the *M*. *cyclopis* mitogenome. As expected, our results show that its organization and gene order are very similar to those of other macaques and phylogeny based on mitogenomes of several macaque species supports a close relationship of *M*. *cyclopis* to *M*. *mulatta mulatta* and *M*. *mulatta lasiota* in the fascicularis group.

## Materials and Methods

### Ethics statement

The macaque used in this study was rescued and adopted in Rescue Center for Endangered Wild Animals, National Pingtung University of Science and Technology (NPUST). This study was approved by Institutional Animal Care and Use Committee of National Pingtung University of Science and Technology Laboratory Animal Center. (Approval Number: NPUST-IACUC-101-082).

The blood samples were collected from 25 Formosan macaques individually which have been kept in the same troop in Pingtung Rescue Center of Wild Endangered Animals for more than 5 years. Only one female macaque was selected for this study after comprehensive examination. This troop that contained all the 25 Formosan macaques were kept in the 30 x 30 m outdoor enclosure which filled with branches, ropes and platforms and were provided with 30kg fresh fruit and 40kg fresh vegetables twice daily. Samples were obtained as part of a comprehensive health screening effort conducted biannually by Pingtung Rescue Center of Wild Endangered Animals and was handled by veterinarians with professional personnel of Pingtung Rescue Center of Wild Endangered Animals.

During blood sample collection, macaques were trapped in a portable cage measuring 80 × 80 × 120 cm separately and sedated with 5 mg/kg ketamine combined with 0.25 mg/kg xylazine intramuscularly. After induction of anesthesia, macaques were incubated and the anesthesia was then maintained by isoflurane inhalation and monitored by SpO2, heart rate and respiration rate continuously. After sample collection, the anesthesia was reversed by 0.2 mg/kg atipamezole and animals were placed in the portable cage separately for fully recovery from anesthesia before being released back into the outdoor enclosure.

All anesthetized macaques were given a complete physical examination, and using universal precautions and sterile technique by veterinarians. The veterinarians and personnel of Pingtung Rescue Center of Wild Endangered Animals monitored and handled all anesthetized macaques during procedure. None of the macaques was sacrificed at the end of the study.

### Sample collection, DNA extraction and sequencing

**Fig 1:**
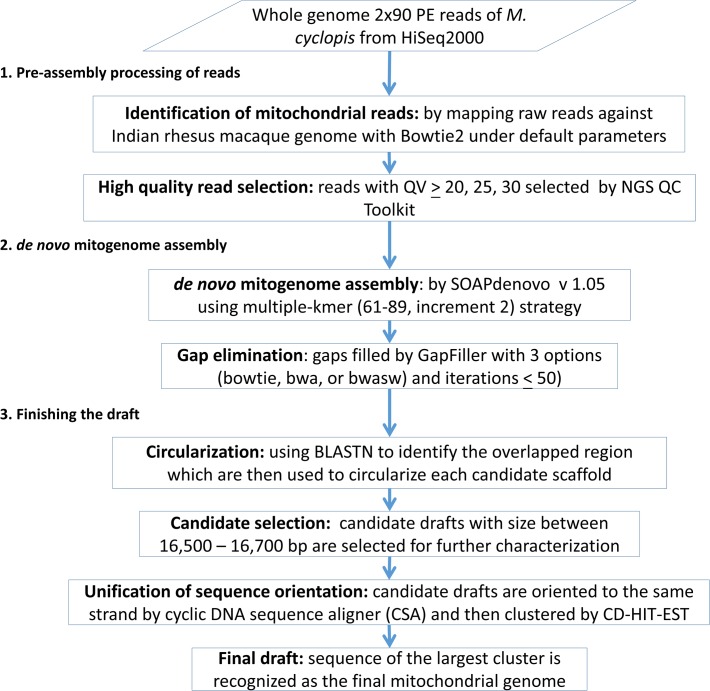
The simple *de novo* mitochondrial genome assembly pipeline for the Taiwanese macaque mitochondrial genome.

Out of 25 macaques, one female *M*. *cyclopis* (NPUST ID: 12060805) from Institute of Wildlife Conservation, National Pingtung University of Science and Technology was used for this study. A total of 10 mL blood was collected by venipuncture of the femoral vein out of which 7 mL blood was aliquotted into Vacutainer vials containing EDTA whereas the remaining blood was centrifuged to extract serum. Serum and whole blood were stored at −20°C and 4°C and later used in further analysis. Genomic DNA was isolated from the peripheral blood cells, sonicated, electrophoresed, and the ~500 bp fragments were isolated with gel excision and used for whole genome sequencing library construction. The library was subjected to 2 x 90 bp paired-end (PE) sequencing by Illumina HiSeq2000 instrument. Followed by reference-assisted *de novo* mitochondrial genome assembly pipeline is shown in Fig 1.

### Pre-assembly processing of reads

To separate the mitochondrial reads of *M*. *cyclopis* we used mitogenome of Indian rhesus macaque (*M*. *mulatta*) [GenBank: NC_005943] as reference and mapped raw reads to reference with Bowtie2 [23] using default parameters. Our method includes reads selection using reference mitogenome, hence we named it as reference-assisted. Mapped read IDs were retrieved from BAM file even if only one mate of paired-end read was mapped. Paired-end (PE) reads were extracted by seqtk (https://github.com/lh3/seqtk) software according to the list extracted from the BAM file. NGS QC Toolkit [24] was used to filter PE reads with QV ≥ 20 for all bases to construct a read pool. This read pool might contain SE reads as the pair might have broken because of lower QV than threshold. Similarly two more read pools were generated with QV ≥ 25 and QV ≥ 30 for all bases. These three read pools were each separated into two pools having 1) only paired-end reads (PE), and 2) both paired-end reads and single-end reads (PE+SE) thereby making six pools in total. This process of constructing six read pools is designed to increase variability. All six read pools were used for *de novo* assembly of the mitogenome. Coverage was estimated using formula: C = (N * L * 2) / G for PE reads, and C = (N * L) / G for SE reads, where C stands for coverage, N is the number of reads in the sequencing library, L is the read length, and G is the size of the target genome.

### *De novo* mitochondrial genome assembly with multiple k-mer strategy

The idea to assemble the mitochondrial genome of Taiwanese macaque was to utilize all possible scaffolds generated by assembler with 15 different k-mer combinations (starting from minimum of 61 to maximum of 89 by increment of 2). In addition, we generated different read pools with corresponding QV threshold for NGS QC Toolkit described in the previous section. The multiple k-mer strategy combines the advantages of the capability of large k-mers to resolve repetitive sequences and the capability of small k-mers to assemble low coverage bases and overcome sequencing errors [25, 26].

This pipeline was used to produce scaffolds from 90 different combinations (15 k-mers on 6 read pools). We used SOAPdenovo v1.05 [27] to obtain scaffolds. Further to eliminate gaps on scaffolds we used GapFiller [28] with three options: bowtie, bwasw and bwa, with a maximum of 50 iterations for sequence convergence.

### Finishing the draft

**Fig 2:**
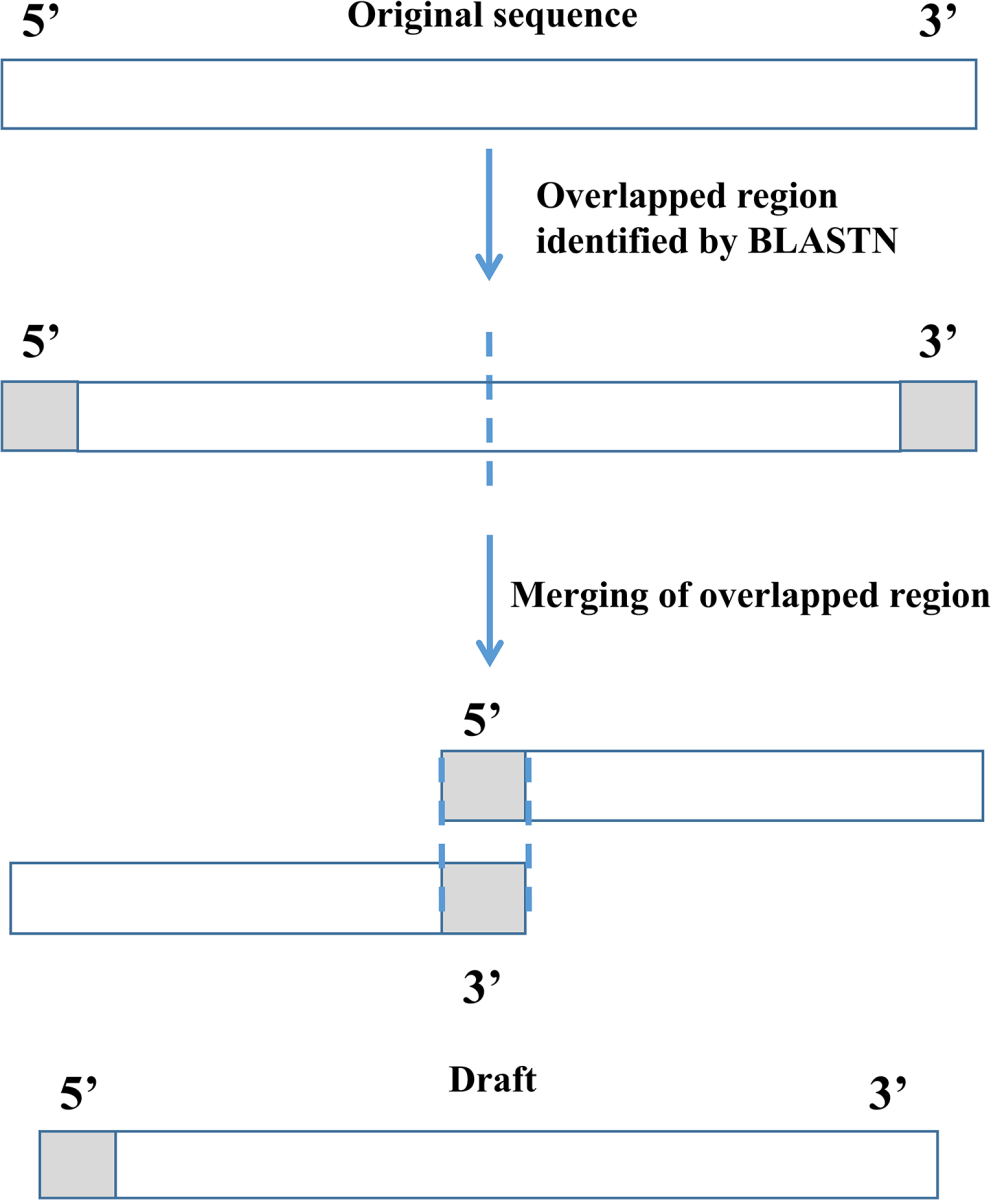
Illustration of checking for boundary overlap to finish the mitochondrial genome. Circular mitochondrial DNA was assembled to scaffolds in linear form each of which exhibited overlapped regions at the ends.

Circular mitochondrial DNA was sequenced in the shotgun library and assembled into a linear sequence, therefore, a repeat (overlapping) region at both the 5’- and 3’-ends should be present [29]. We used BLASTN from NCBI BLAST+ [30] to determine the overlapped region for each scaffold and circularize the scaffold by merging the overlapped regions (Fig 2). Based on the size of existing mitogenomes of macaque species, scaffolds with sizes ranging between 16,500 bp and 16,700 bp were retained as mitogenome candidates.

All mitogenome candidates assembled by *de novo* mitogenome assembly pipeline, were collected. The orientation of candidate mitogenomes was unified by CSA (cyclic DNA sequence aligner) [31] and then compared with each other using CD-HIT-EST in CD-HIT package [32, 33] with the consideration of forward and reversed strands. Sequence represented by the cluster with the largest number of identical sequences is concluded as mitochondrial genome of *M*. *cyclopis*.

### Mitochondrial genome annotation and sequence comparison

To identify the boundaries of protein-coding genes, tRNA genes, and rRNA genes for annotation of the *M*. *cyclopis* mitogenome, MITOS [34] and DOGMA [35] were used. Mitogenome and gene boundaries, both were validated using BLASTN by comparing gene annotations of Indian rhesus macaque and with related mtDNA fragments available in GenBank. For protein-coding genes, sequences were translated by Sequin software and verified by aligning the translated sequence against the UniProt/TrEMBL database. The circular map of the mitochondrial genome was generated by CGView [36]. Repeat regions were detected by RepeatMasker (http://www.repeatmasker.org/) with rmblast. Clustal Omega [37] was employed for multiple sequence alignment on the mitogenome and DNA fragments respectively.

### Comparative phylogenetic analysis

To study comparative phylogenetics of macaques, each mitochondrial genome was aligned by unifying starting position with respect to mitogenome of Indian rhesus macaque [GenBank: NC_005943]. Phylogenetic relationships among the macaques based on the complete mitogenome were estimated with Clustal Omega. Ambiguously aligned positions were removed by using Gblocks v0.91b [38] under default settings. We used MrBayes v3.2.3 [39], one of hierarchical Bayesian inference (BI) tools, to estimate phylogeny. Each search was run simultaneous Markov chains for 40000 generations, sampling every 1000 generations. Generations sampled before the chain reached stationarity (“burn-in”) were discarded. Phylogenetic analysis has been demonstrated on the complete mitochondrial genome using the *Homo sapiens* mitogenome [GenBank: NC_012920] as outgroup. Tree was visualized with FigTree v1.4.2 (http://tree.bio.ed.ac.uk/software/figtree/). In addition, pairwise sequence identity was determined by LAGAN [40] to analyse sequence-level variation.

## Results and Discussion

### The completed *M*. *cyclopis* mitochondrial genome

A total of 574,495,990 2 x 90 bp paired-end (PE) reads were generated by an Illumina HiSeq2000 sequencer from the whole genome shotgun sequencing library of *M*. *cyclopis*. Among those, 222,251 raw reads were mapped to the Indian rhesus reference mitogenome (S1 Dataset). The overall alignment rate is 0.04% and the coverage is 2,265X±578X with median of 2,304X. Coverage was estimated assuming mitogenome to be around 16,500bp. Read pools generated after QV based selection are summarised in Table 1. Six read pools were then processed to retrieve draft mitogenome candidates with our assembly pipeline. No ambiguous bases were found in these draft mitogenomes.

**Table 1:** Library statistics of quality reads and coverage information.

Quality score cut-off	Paired-End (PE) reads	Single-End (SE) reads	Total bases	Coverage(average)
QV ≥ 20	64,998	76,644	18,597,600	1,127
QV ≥ 25	39,254	78,261	14,109,210	855
QV ≥ 30	11,241	57,062	7,158,960	434

By comparing these candidates it was revealed that largest cluster contained 7 identical sequences (4 forward strand forms and 3 reversed strand forms), while other two clusters contained only 1 sequence each. Thus, the sequence of the largest cluster was recognized as the complete sequence of *M*. *cyclopis* mitogenome. The *M*. *cyclopis* mitogenome (GenBank accession number KM023192) is a double-stranded nucleotide sequence of 16,563 bp, falling within the size range of other published macaque mitogenome sequences (Table 2) including Asian and Northern African macaques. The sizes of macaque mitogenomes sequenced to date range from 16,540 bp in *M*. *thibetana* to 16,586 bp in *M*. *sylvanus*; the size of the Taiwanese macaque mitogenome (16,563) is closest to that of *M*. *mulatta* (16,564), *M*. *fuscata* (16,565) and *M*. *mulatta lasiota* (16,561).

**Table 2:** Comparison of mitochondrial size between macaque species.

Species	Size (bp)	GenBank accession number	Reference
*M*. *mulatta*	16,555	KJ567051	[6]
*M*. *mulattalasiota*	16,561	KF830702	[7]
*M*. *fuscata*	16,565	NC_025513 / KM401548	[12]
*M*. *mulatta*	16,564	JQ821843	[5]
*M*. *mulatta*	16,564	NC_005943	[4]
*M*. *mulatta*	16,564	KJ567053	[6]
*M*. *arctoides*	16,559	KM360179	[11]
*M*. *arctoides*	16,558	NC_025201 / KJ567055	[6]
*M*. *fascicularis*	16,569	KJ567052	[6]
*M*. *fascicularis*	16,571	KF305937	[8]
*M*. *fascicularis*	16,575	NC_012670	Yi, et al. 2009, unpublished
*M*. *thibetana*	16,540	NC_011519	[9]
*M*. *thibetana*	16,539	KJ567056	[6]
*M*. *assamensis*	16,542	NC_023795 / KF990122	[10]
*M*. *tonkeana*	16,560	NC_025222 / KJ567058	[6]
*M*. *silenus*	16,541	NC_025221 / KJ567057	[6]
*M*. *sylvanus*	16,586	NC_002764	[49]
*M*. *sylvanus*	16,585	KJ567054	[6]
*M*. *cyclopis*	16,563	KM023192	This report

### Genome organization and gene arrangement

**Fig 3:**
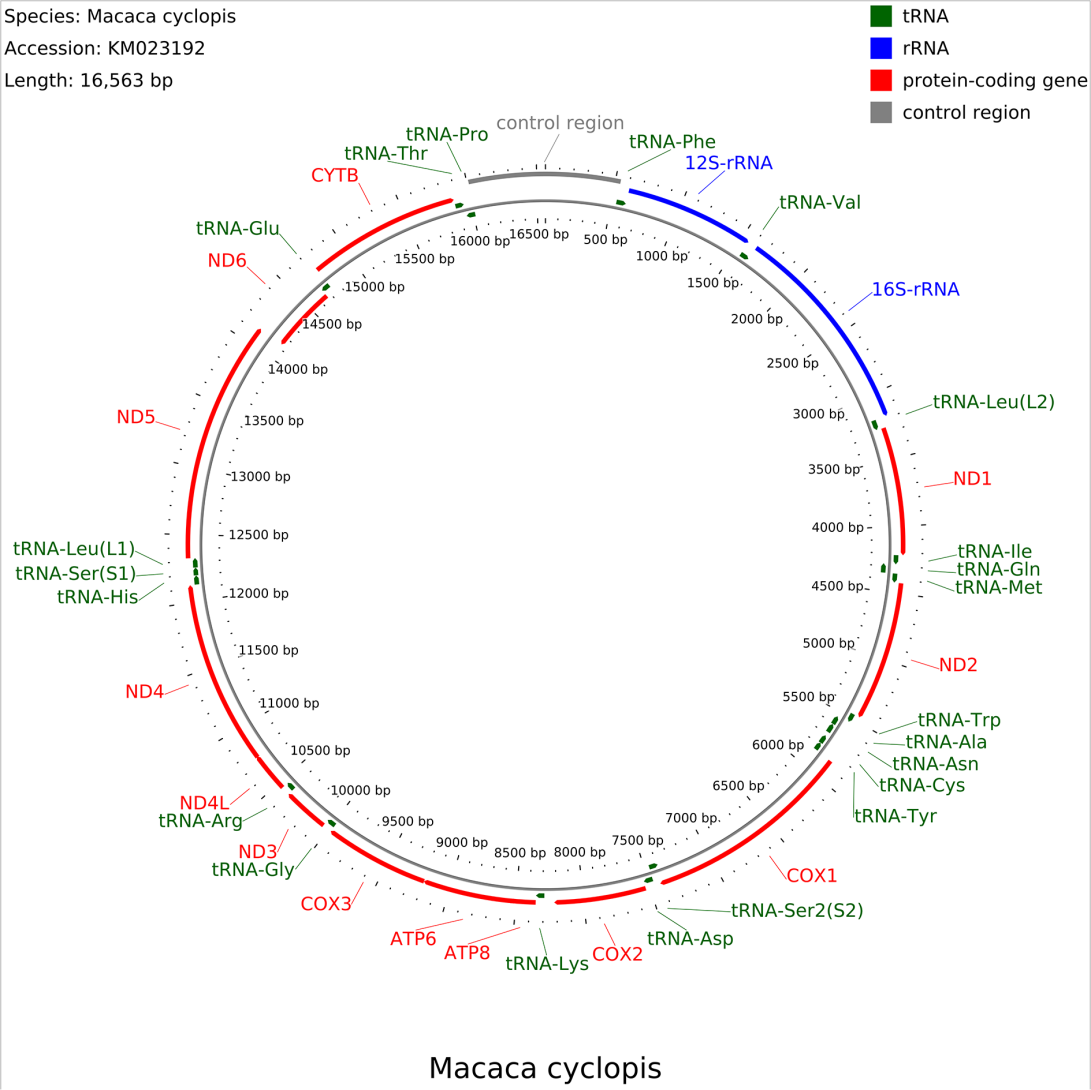
The organization of the mitogenome of *M*. *cyclopis*. The outer and the inner circles represent the H- and L-strands, respectively. The tRNA, rRNA, protein-coding genes and control region are represented in green, red, blue and gray, respectively.

The complete organization of the mitochondrial genome of *M*. *cyclopis* is shown in Fig 3. It contains 13 protein-coding genes (ND1-6, ND4L, COX 1–3, ATP6, ATP8, CYTB), 2 ribosomal RNAs (12S and 16S), 22 transfer RNAs and a control region (Table 3). The heavy strand (H-strand) contains 2 ribosomal RNAs, 14 transfer RNAs and 12 protein-coding genes while the light strand (L-strand) contains 8 transfer RNAs and one protein-coding gene. The origin of light strand replication, O_L_, located between tRNA-Asn and tRNA-Cys, is 32 bp in length. In addition, there are five overlaps between adjacent genes out of which three are same strand overlap. Among same strand overlaps there is a 46 bp overlap between ATP8 and ATP6, a 1 bp overlap between ATP6 and COX3, and a 7 bp overlap between ND4 and ND4L. We identified a 3 bp overlap between tRNA-Ile and tRNA-Gln and a 28 bp overlap between COX1 and tRNA-Ser, both of gene pairs lie on opposite strands.

**Table 3:** Summary of the mitochondrial genome organization of *M*. *cyclopis*.

Name	Position (Start-End)	Strand	Length	# of AA	Anti-codon	Inferred initiation codon	Inferred termination codon	Intergenic spacer^1^
tRNA-Phe		H	72		GAA			0
tRNA-Phe	538–609	H	72		GAA			0
rrnS (12S)	610–1556	H	947					0
tRNA-Val	1557–1625	H	69		TAC			0
rrnL (16S)	1626–3184	H	1559					0
tRNA-Leu (L2)	3185–3259	H	75		TAA			2
ND1	3262–4216	H	955	318		ATG	T—	0
tRNA-Ile	4217–4285	H	69		GAT			-3
tRNA-Gln	4283–4354	L	72		TTG			1
tRNA-Met	4356–4423	H	68		CAT			0
ND2	4424–5465	H	1042	347		ATT	T—	0
tRNA-Trp	5466–5532	H	67		TCA			7
tRNA-Ala	5540–5608	L	69		TGC			1
tRNA-Asn	5610–5682	L	73		GTT			32
tRNA-Cys	5715–5783	L	69		GCA			0
tRNA-Tyr	5784–5847	L	64		GTA			4
COX1	5852–7420	H	1569	522		ATG	TAA	-28
tRNA-Ser (S2)	7393–7461	L	69		TGA			3
tRNA-Asp	7465–7532	H	68		GTC			1
COX2	7534–8217	H	684	227		ATG	TAA	73
tRNA-Lys	8291–8353	H	63		TTT			1
ATP8	8355–8561	H	207	68		ATG	TAA	-46
ATP6	8516–9196	H	681	226		ATG	TAA	-1
COX3	9196–9979	H	784	261		ATG	T—	0
tRNA-Gly	9980–10047	H	68		TCC			0
ND3	10048–10393	H	346	115		ATT	T—	0
tRNA-Arg	10394–10458	H	65		TCG			0
ND4L	10459–10755	H	297	98		ATG	TAA	-7
ND4	10749–12126	H	1378	459		ATG	T—	0
tRNA-His	12127–12195	H	69		GTG			0
tRNA-Ser (S1)	12196–12254	H	59		GCT			0
tRNA-Leu (L1)	12255–12325	H	71		TAG			0
ND5	12326–14137	H	1812	603		ATA	TAA	0
ND6	14138–14665	L	528			ATG	AGG	0
tRNA-Glu	14666–14734	L	69		TTC			4
CYTB	14739–15879	H	1141	380		ATG	T—	0
tRNA-Thr	15880–15943	H	64		TGT			1
tRNA-Pro	15945–16012	L	68		TGG			0

^1^Intergenic spacer: numbers indicate nucleotides separating two adjacent genes. Negative numbers indicate overlapping nucleotides

### Protein coding genes (PCGs)

The 13 protein-coding genes of the *M*. *cyclopis* mitochondrial genome include 7 NADH dehydrogenase subunits (ND1-6, ND4L), 3 cytochrome c oxidase subunits (COX1-3), 2 ATP synthase subunits (ATP6, ATP8), and cytochrome b (CYTB). Ten of 13 protein-coding genes were initiated with ATG, while ND2 and ND3 shared ATT as their start codon, and ND5 utilized ATA. Six protein-coding genes (COX1, COX2, ATP8, ATP6, ND4L, ND5) ended with TAA, whereas ND6 terminated with AGG. Remaining six genes that comprise ND1, ND2, COX3, ND3, ND4 and CYTB were found to have incomplete stop codons (T—).

### Ribosomal RNA and transfer RNA

Taiwanese macaque mtDNA contains the small unit rRNA (12S rRNA) and large unit rRNA (16S rRNA) genes. They are located between tRNA-Phe and tRNA-Leu (L2) genes, and separated from each other by the tRNA-Val gene. The sizes of the two ribosomal RNAs are 947 bp and 1,209 bp, respectively. There are 22 tRNA genes, with sizes ranging from 59 bp of tRNA-Ser to 75 bp of tRNA-Leu (L2), that include two more tRNAs that code for leucine and serine. The mitochondrial WANCY tRNA-gene cluster, located between ND2 and COX1, is 383 bp in length.

### Control region (hypervariable region, non-coding regions)

The control region (D-loop) of the mitogenomes of macaques, size of which varies from 1,083 bp in *M*. *mulatta lasiota* to 1,100 bp in *M*. *fascicularis* [GenBank: KF305937], is located between tRNA-Pro and tRNA-Phe. This region is 1,088 bp long in mitogenome of *M*. *cyclopis*. RepeatMasker identified a (TCGTACA)n repeat within 43 bp located in nps 282–322 of the D-loop.

The control region of the Taiwanese macaque exhibits a higher GC ratio than other macaque species of the fascicularis group (Table 4). Within this sequence, we identified the central domain, extended termination associated sequences (ETAS) domain and conserved sequence blocks (CSB) domain based on previous studies [9, 41] with multiple sequence alignment of the D-Loop of 19 macaques. The central domain is located in the position 364–686. CSB1, CSB2 and CSB3 are located in nps 763–787, 849–864 and 892–910, respectively. The ETAS1 and ETAS2 are near the 5’ end of the control region, at nps 57–113 and 275–334, respectively.

**Table 4:** Characteristics of the D-loop/control region of macaque species.

Species	GenBank Acc.	Size (bp)	A		C		G		T		N	G+C (%)	CpG
			bp	%	bp	%	bp	%	bp	%			
*M*. *mulatta*	JQ821843	1085	321	29.59%	341	31.43%	135	12.44%	288	26.54%	0	43.87%	58
*M*. *fascicularis*	KF305937	1100	326	29.64%	344	31.27%	141	12.82%	289	26.27%	0	44.09%	66
*M*. *mulatta lasiotus*	KF830702	1083	320	29.55%	337	31.12%	138	12.74%	288	26.59%	0	43.86%	58
*M*. *mulatta*	KJ567051	1083	322	29.73%	332	30.66%	136	12.56%	293	27.05%	0	43.21%	54
*M*. *fascicularis*	KJ567052	1096	330	30.11%	336	30.66%	133	12.14%	297	27.10%	0	42.79%	56
*M*. *mulatta*	KJ567053	1085	319	29.40%	339	31.24%	136	12.53%	290	26.73%	1	43.78%	58
*M*. *sylvanus*	KJ567054	1094	322	29.43%	357	32.63%	140	12.80%	275	25.14%	0	45.43%	58
*M*. *thibetana*	KJ567056	1070	323	30.19%	341	31.87%	135	12.62%	271	25.33%	0	44.49%	56
*M*. *arctoides*	KM360179	1083	317	29.27%	353	32.59%	144	13.30%	269	24.84%	0	45.89%	74
*M*. *sylvanus*	NC_002764	1095	322	29.41%	354	32.33%	141	12.88%	278	25.39%	0	45.21%	60
*M*. *mulatta*	NC_005943	1085	321	29.59%	341	31.43%	135	12.44%	288	26.54%	0	43.87%	58
*M*. *thibetana*	NC_011519	1091	329	30.16%	343	31.44%	138	12.65%	281	25.76%	0	44.09%	56
*M*. *fascicularis*	NC_012670	1095	329	30.05%	339	30.96%	138	12.60%	289	26.39%	0	43.56%	58
*M*. *assamensis*	NC_023795	1091	326	29.88%	340	31.16%	140	12.83%	285	26.12%	0	44.00%	58
*M*. *arctoides*	NC_025201	1081	318	29.42%	342	31.64%	143	13.23%	278	25.72%	0	44.87%	72
*M*. *silenus*	NC_025221	1088	316	29.04%	334	30.70%	143	13.14%	295	27.11%	0	43.84%	54
*M*. *tonkeana*	NC_025222	1088	320	29.41%	342	31.43%	138	12.68%	288	26.47%	0	44.12%	62
*M*. *fuscata*	NC_025513	1088	320	29.41%	345	31.71%	141	12.96%	282	25.92%	0	44.67%	56
*M*. *cyclopis*	KM023192	1088	316	29.04%	340	31.25%	142	13.05%	290	26.65%	0	44.30%	62

Three datasets of mtDNA fragments associated with the Taiwanese macaque have previously been deposited in GenBank. These dataset were used for validating the complete mitochondrial genome of *M*. *cyclopis* and the alignment results in terms of identity, mismatch and gap are shown in Table 5. In a study by Smith and colleagues, out of 1,053 samples, 53 samples (DQ373370~DQ373422) [18] were from Taiwanese macaques. They sequenced 835 bp long mtDNA fragment containing one seventh of CYTB, tRNA-Pro, tRNA-Thr and HVS-1(first hypervariable segment of control region) of the fascicularis group of macaque species for the analysis of mitochondrial DNA variation. Sequence identity ranges from 98.66% to 100% without gap was validated in the coding region of CYTB, tRNA-Pro, and tRNA-Thr. We compared previously sequenced 8 fragments (called 8 samples) from different studies (Table 6) with similar region in mitogenome of *M*. *cyclopis*. These include 12S rRNA, 16S rRNA, tRNA-Val, tRNA-Glu, COX1-3 and CYTB (AF424944.1, AF424945.1 [42]; AY685836.1, AY685877.1, AY685795.1, AY685786.1, AY685713.1 [16] and AJ304499.1 (unpublished)). The sequence identity ranges from 98.66% to 100% with full-length alignment of query sequences without gap. 73 control region-containing fragments (called 73 samples) that include AB261600.1 [43], AY014865.1 ~ AY014877.1, AY016337.1 [44], AY682594.1 [45], AY878873.1 ~ AY878925.1, DQ143984.1 ~ DQ143987.1 [13] were also compared with the control region of *M*. *cyclopis* and 91.62% to 99.65% sequence identity was found. In summary, variation in the control region among individuals is obvious in the alignment results with far more mismatches and gaps than occur in the coding region.

**Table 5:** Results of sequence alignment of the complete *M*. *cyclopis* mitochondrial genome against the three existing datasets of *M*. *cyclopis* partial mitochondrial fragments.

Dataset	Type^1^	Identity			Mismatch		Gap	
		Overall	Min	Max	Min	Max	Min	max
53 samples	Overall	96.22	95.45	97.49	20	37	0	1
	Coding region	99.20	98.66	100.00	0	4	0	0
	Control region	94.56	93.68	96.10	20	33	0	1
8 samples	Coding region	99.57	98.66	100.00	0	7	0	0
73 samples	Control region	94.87	91.62	99.65	2	45	0	4

^1^Overall: whole mitogenome as a subject sequence in BLASTN. Coding region: the coding region of the mitogenome, starting with tRNA-Phe and ending with tRNA-Pro, as a subject sequence in BLASTN. Control region: the control region of the mitogenome as a subject sequence in BLASTN.

**Table 6:** List of sequenced fragments of *M*. *cyclopis* mitochondrial genome.

GenBank accession number	Description	Reference
AY685877.1	*M*. *cyclopis* isolate Taiwan 12S ribosomal RNA gene, partial sequence; mitochondrial	[16]
AF424944.1	*M*. *cyclopis* isolate 1 12S ribosomal RNA gene, partial sequence; tRNA-Val gene, complete sequence; and 16S ribosomal RNA gene, partial sequence; mitochondrial genes for mitochondrial products	[42]
AF424945.1	*M*. *cyclopis* isolate 2682 12S ribosomal RNA gene, partial sequence; tRNA-Val gene, complete sequence; and 16S ribosomal RNA gene, partial sequence; mitochondrial genes for mitochondrial products	[42]
AY685713.1	*M*. *cyclopis isolate Taiwan cytochrome oxidase subunit I gene*, *partial cds; mitochondrial*	[16]
AY685786.1	*M*. *cyclopis isolate Taiwan cytochrome oxidase subunit II gene*, *partial cds; mitochondrial*	[16]
AY685795.1	*M*. *cyclopis isolate Taiwan cytochrome oxidase subunit III gene*, *partial cds; mitochondrial*	[16]
AY685836.1	*M*. *cyclopis* isolate Taiwan tRNA-Glu gene, partial sequence; and cytochrome b gene, partial cds; mitochondrial	[16]
AJ304499.1	*M*. *cyclopis* mitochondrial partial cytb gene for cytochrome b	Lee, unpublished (2000)

### Comparative phylogenetic analysis

**Fig 4:**
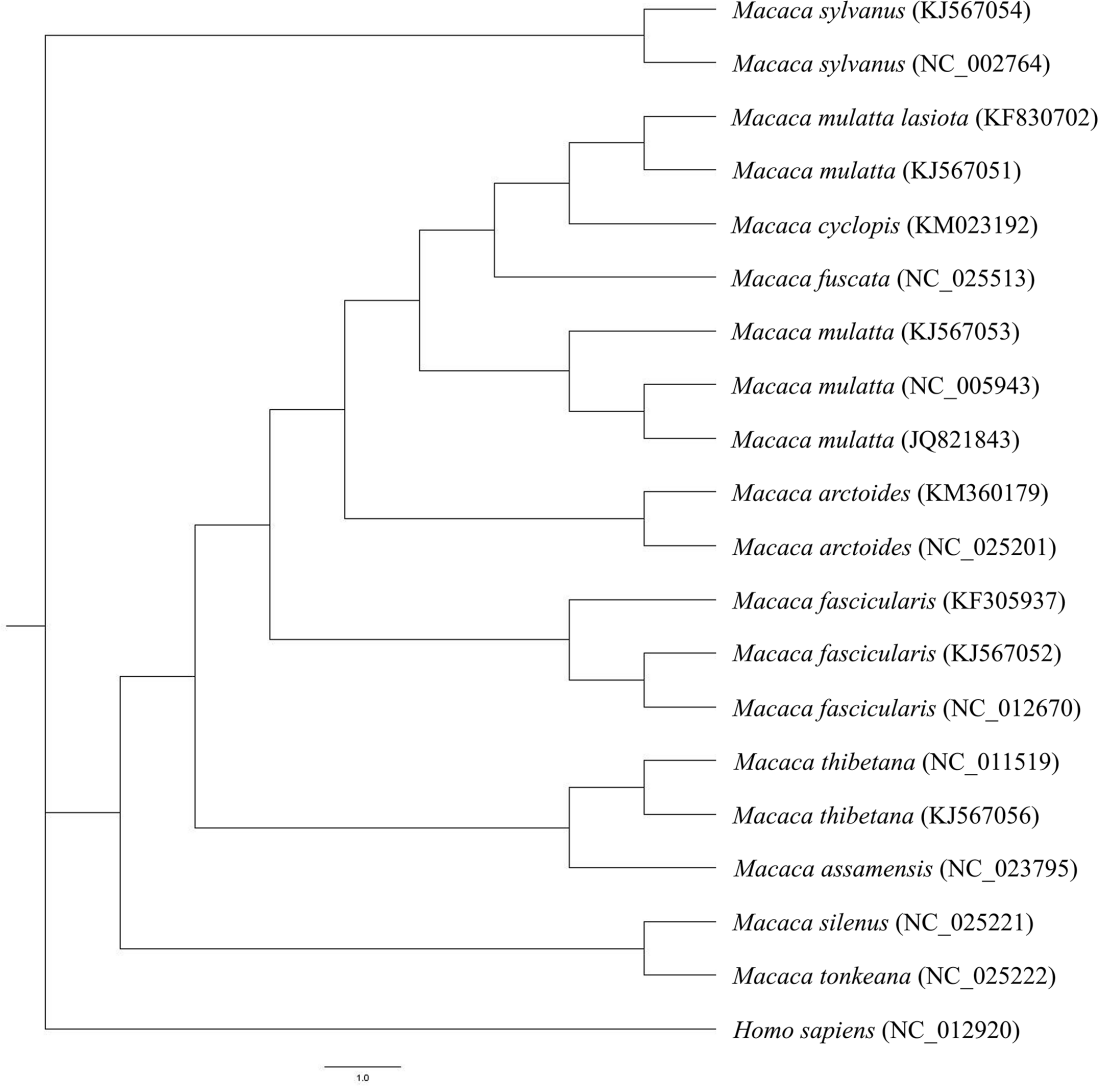
Phylogenetic analysis based on whole mitochondrial genome sequence. The evolutionary history was inferred by MrBayes. The scale is 1.0 expected changes per site. The outgroup for evolutionary analysis is *Homo sapiens* mitochondrion with GenBank acc. NC_012920.

We compare the mitochondrial genome of 19 different macaques including *M*. *cyclopis* and created phylogenetic trees by applying Bayesian method (Fig 4). Pair-wise sequence identity between macaque mitochondrial genomes indicate that *M*. *cyclopis* is phylogenetically closer to *M*. *mulatta lasiota* than to *M*. *mulatta mulatta*as well (Table 7). Our results resemble to those of previous reports by Balasubramaniam et al. [2], Chu et al. [13], Zhao et al. [46], and Smith et al. [47].

**Table 7:** Pair-wise sequence similarity between macaques by LAGAN.

		KF830702	NC_025513	JQ821843	NC_005943	KJ567053	KM360179	NC_025201	KJ567052	KF305937	NC_012670	NC_011519	KJ567056	NC_023795	NC_025222	NC_025221	KJ567054	NC_002764	KM023192
		*M*. *mulatta lasiotus*	*M*. *fuscata*	*M*. *mulatta*	*M*. *mulatta*	*M*. *mulatta*	*M*. *arctoides*	*M*. *arctoides*	*M*. *fascicularis*	*M*. *fascicularis*	*M*. *fascicularis*	*M*. *thibetana*	*M*. *thibetana*	*M*. *assamensis*	*M*. *tonkeana*	*M*. *silenus*	*M*. *sylvanus*	*M*. *sylvanus*	*M*. *cyclopis*
KJ567051	*M*. *mulatta*	99.26%	96.41%	95.72%	95.72%	95.71%	93.03%	92.95%	92.90%	92.88%	92.58%	91.55%	91.57%	91.41%	90.39%	90.21%	89.66%	89.60%	96.71%
KF830702	*M*. *mulatta lasiotus*	-	96.35%	95.71%	95.71%	95.71%	92.91%	92.84%	92.99%	92.93%	92.62%	91.59%	91.60%	91.37%	90.36%	90.25%	89.69%	89.62%	96.64%
NC_025513	*M*. *fuscata*	-	-	95.57%	95.57%	95.59%	92.95%	92.90%	92.75%	92.65%	92.40%	91.28%	91.29%	91.18%	90.20%	90.16%	89.57%	89.47%	96.46%
JQ821843	*M*. *mulatta*	-	-	-	100.00%	99.93%	92.93%	92.90%	92.86%	92.93%	92.62%	91.43%	91.45%	91.24%	90.17%	90.28%	89.44%	89.36%	95.62%
NC_005943	*M*. *mulatta*	-	-	-	-	99.93%	92.93%	92.90%	92.86%	92.93%	92.62%	91.43%	91.45%	91.24%	90.17%	90.28%	89.44%	89.36%	95.62%
KJ567053	*M*. *mulatta*	-	-	-	-	-	92.92%	92.89%	92.86%	92.93%	92.60%	91.41%	91.43%	91.22%	90.17%	90.25%	89.45%	89.37%	95.62%
KM360179	*M*. *arctoides*	-	-	-	-	-	-	98.75%	92.46%	92.52%	92.08%	91.36%	91.36%	91.18%	90.30%	90.28%	89.37%	89.30%	93.01%
NC_025201	*M*. *arctoides*	-	-	-	-	-	-	-	92.49%	92.53%	92.02%	91.25%	91.28%	91.08%	90.14%	90.27%	89.50%	89.42%	93.01%
KJ567052	*M*. *fascicularis*	-	-	-	-	-	-	-	-	97.98%	97.68%	91.60%	91.63%	91.37%	90.20%	90.07%	89.68%	89.59%	92.88%
KF305937	*M*. *fascicularis*	-	-	-	-	-	-	-	-	-	96.41%	91.62%	91.64%	91.43%	90.19%	90.27%	89.74%	89.67%	92.88%
NC_012670	*M*. *fascicularis*	-	-	-	-	-	-	-	-	-	-	91.34%	91.32%	91.06%	90.05%	89.95%	89.56%	89.49%	92.56%
NC_011519	*M*. *thibetana*	-	-	-	-	-	-	-	-	-	-	-	99.89%	98.04%	89.65%	89.83%	89.21%	89.14%	91.47%
KJ567056	*M*. *thibetana*	-	-	-	-	-	-	-	-	-	-	-	-	98.05%	89.62%	89.82%	89.23%	89.15%	91.47%
NC_023795	*M*. *assamensis*	-	-	-	-	-	-	-	-	-	-	-	-	-	89.46%	89.72%	89.17%	89.11%	91.40%
NC_025222	*M*. *tonkeana*	-	-	-	-	-	-	-	-	-	-	-	-	-	-	91.88%	89.36%	89.29%	90.33%
NC_025221	*M*. *silenus*	-	-	-	-	-	-	-	-	-	-	-	-	-	-	-	89.51%	89.44%	90.23%
KJ567054	*M*. *sylvanus*	-	-	-	-	-	-	-	-	-	-	-	-	-	-	-	-	99.88%	89.76%
NC_002764	*M*. *sylvanus*	-	-	-	-	-	-	-	-	-	-	-	-	-	-	-	-	-	89.67%

**Fig 5:**
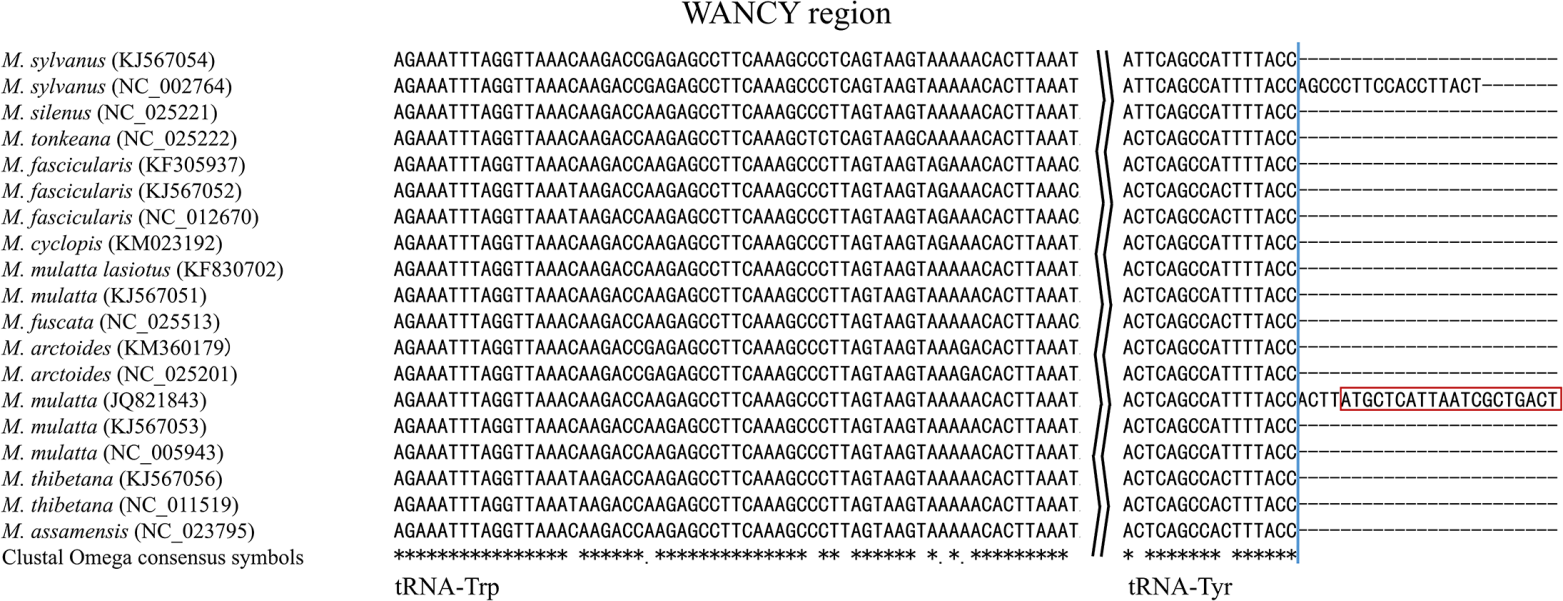
Insertion in tRNA-Tyr of the WANCY tRNA-gene cluster. Multiple sequence alignment of the WANCY tRNA-gene cluster identified a 17 bp insertion at the 3’-end of tRNA-Tyr specific to *M*. *sylvanus* and a 24 bp insertion at the 3’-end of tRNA-Tyr specific to *M*. *mulatta*. The red box showed the overlap region between tRNA-Tyr and COX1. The blue line showed the 3’-end boundary of tRNA-Tyr while checking with tRNAscan-SE and MITOS.

Multiple sequence alignment for the region of tRNA-Phe and tRNA-Pro (excluding control region) of the macaques under study revealed variations in the following regions. 17 and 24 bp long different insertions after tRNA-Tyr in WANCY region in *M*. *sylvanus* and *M*. *mulatta* respectively (Fig 5). There is a 20 bp overlap region between tRNA-Tyr and COX1 identified in *M*. *mulatta* (JQ821843). The 3’-end boundary of tRNA-Tyr is the same among all macaques. In our analysis with tRNAscan-SE [48] and MITOS, we found these two insertions and concluded them as result of annotation conflict. In addition, pairwise comparison with the same species from different publications this observation was confirmed. For *M*. *sylvanus*, we compared barbary macaque of GenBank acc. NC_002764 with that of GenBank acc. KJ567054. For *M*. *mulatta*, we compared Indian rhesus macaque of GenBank JQ821843 with both of them of GenBank acc. KJ567053 and NC_005943 respectively.

**Fig 6:**
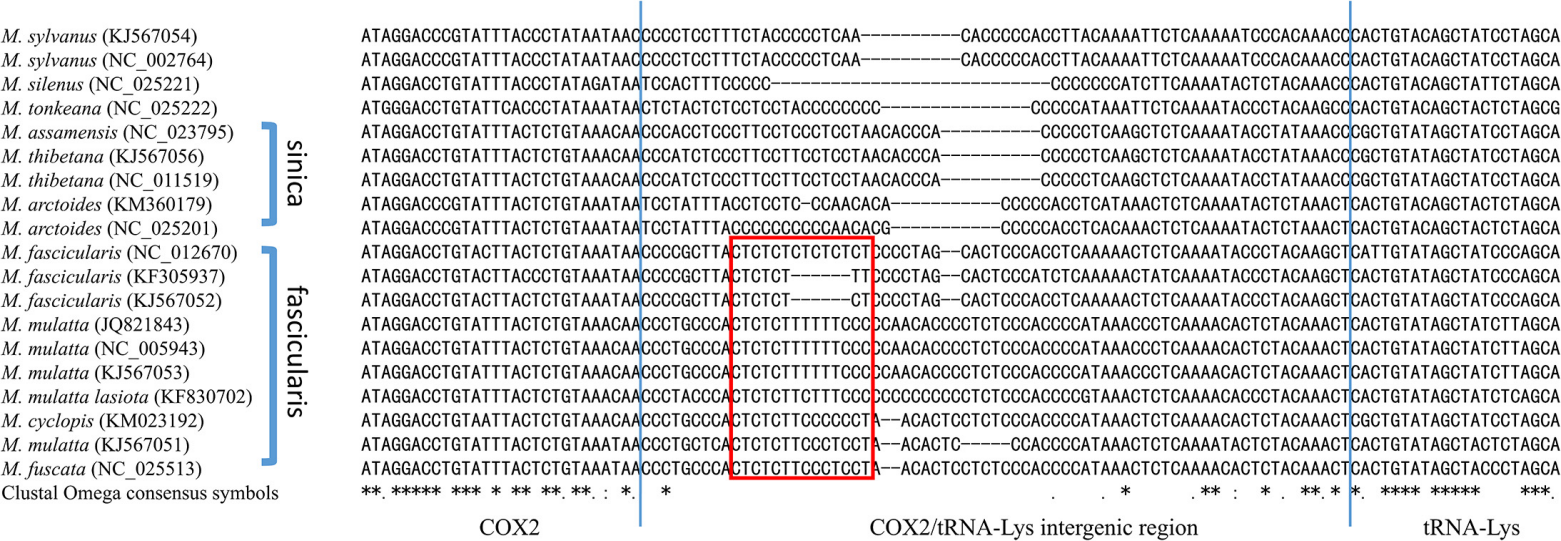
COX2/tRNA-Lys intergenic region. A short CT repeat region in red box is present in the fascicularis group but absent in species of the sinica group. The blue line represents the boundary of COX2 and tRNA-Lys respectively. In sequence alignment, * (asterisk) character indicates positions which are fully conserved.

The intergenic region between COX2 and tRNA-Lys is the short non-coding region in the mitochondrial genome (Fig 6). This intergenic region is the second largest noncoding region in macaque mitogenomes. Comparison with the multiple sequence alignment of macaques, a short CT repeat region found in the fascicularis group but is absent in species of the sinica group.

**Fig 7:**
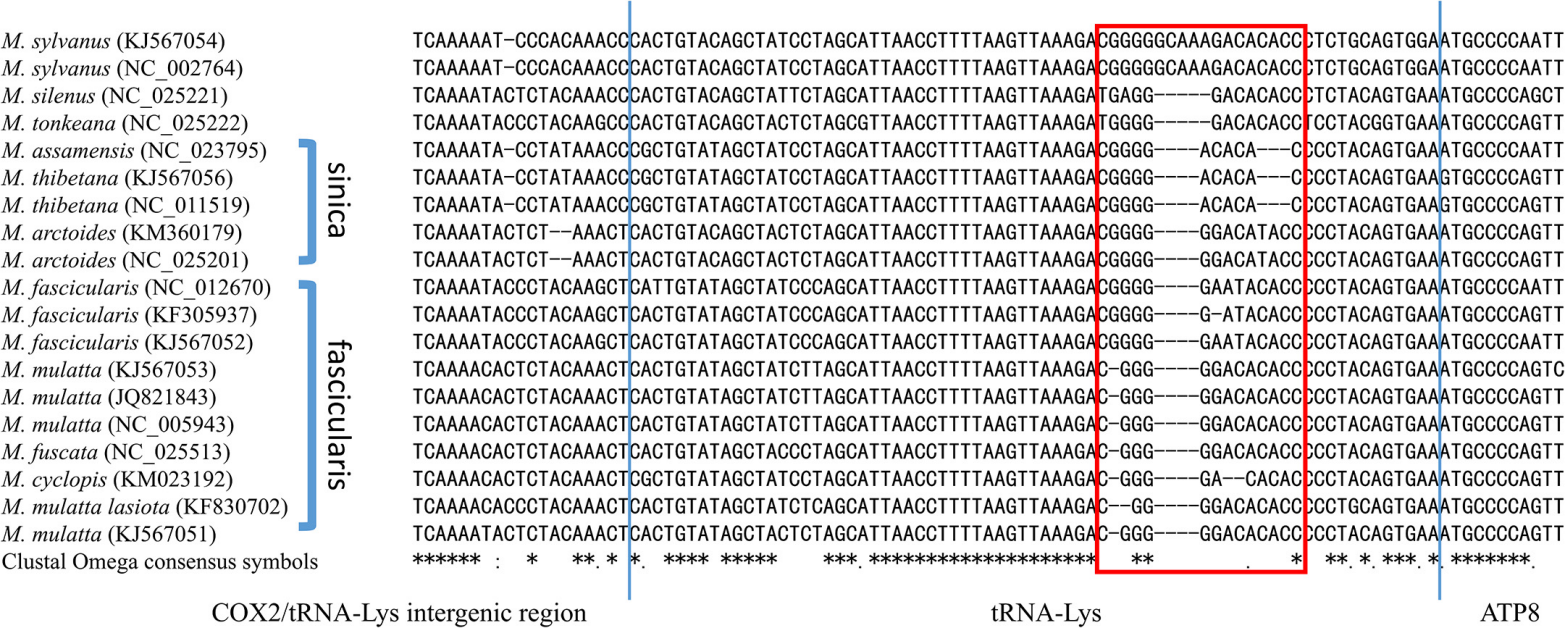
Length variation in tRNA-Lys. The less conserved region (red box) in the 3’-end of tRNA-Lys was explored by multiple sequence alignment. The blue line represents the boundary of 5’-end and 3’-end of tRNA-Lys respectively. In sequence alignment, * (asterisk) character indicates positions which are fully conserved.

We have identified a less conserved region in 3’-end of tRNA-Lys by multiple sequence alignment (Fig 7). Among all tRNA genes, tRNA-Lys gene exhibits a length variation among different macaque species.

Most macaques use ATT/ATG/ATA as the start codon for their mitogenome protein-coding genes (Table 8). Exceptions include ND1 and ATP8 of *M*. *thibetana* and ND1 of *M*. *assamensis*, which use GTG as the start codon. Comparison of the tRNAs of the Taiwanese macaque with those of its closest relatives, the Indian and Chinese rhesus macaques, revealed that only tRNA-Ala and tRNA-Arg are identical among all three macaques.

**Table 8:** Comparison of start and stop codons of protein-coding genes of seven species/subspecies of macaques.

Species	*M*. *mulatta*	*M*. *mulatta lasiotus*	*M*. *fuscata*	*M*. *mulatta*	*M*. *mulatta*	*M*. *mulatta*	*M*. *arctoides*	*M*. *arctoides*	*M*. *fascicularis*	*M*. *fascicularis*	*M*. *fascicularis*	*M*. *thibetana*	*M*. *thibetana*	*M*. *assamensis*	*M*. *tonkeana*	*M*. *silenus*	*M*. *sylvanus*	*M*. *sylvanus*	*M*. *cyclopis*
GenBank acc.	KJ567051	KF830702	NC_025513	JQ821843	NC_005943	KJ567053	KM360179	NC_025201	KJ567052	KF305937	NC_012670	NC_011519	KJ567056	NC_023795	NC_025222	NC_025221	KJ567054	NC_002764	KM023192
ND1	ATG / T—	ATG / T—	ATG / TAA	ATG / T—	ATG / TAG	ATG / T—	ATG / T—	ATG / T—	ATG / T—	ATG / T—	ATG / T—	GTG / T—	GTG / T—	GTG / T—	ATG / T—	ATG / T—	ATG / T—	ATG / TAG	ATG / T—
ND2	ATT / T—	ATT / T—	ATT / TAA	ATT / T—	ATT / TAG	ATT / T—	ATT / T—	ATT / T—	ATT / T—	ATT / T—	ATT / T—	ATT / T—	ATT / T—	ATT / T—	ATT / T—	ATT / T—	ATT / T—	ATT / TAG	ATT / T—
COX1	ATG / TAA	ATG / TAA	ATG / TAA	ATG / TAA	ATG /	ATG / TAA	ATG / TAA	ATG / TAA	ATG / TAG	ATG / TAG	ATG / T—	ATG /	ATG / TAG	ATG / TAG	ATG / TAA	ATG / TAA	ATG / TAG	ATG / G—	ATG / TAA
COX2	ATG / TAA	ATG / TAA	ATG / TAA	ATG / TAA	ATG / TAA	ATG / TAA	ATG / TAA	ATG / TAA	ATG / TAA	ATG / TAA	ATG / TAA	ATG / TAA	ATG / TAA	ATG / TAA	ATG / TAA	ATG / TAG	ATG / TAA	ATG / TAA	ATG / TAA
ATP8	ATG / TAA	ATG / TAA	ATG / TAA	ATG / TAA	ATG / TAA	ATG / TAA	ATG / TAA	ATG / T—	ATG / T—	ATG / TAA	ATG / TAA	GTG / TAA	GTG / T—	ATG / TAA	ATG / TAA	ATG / TAA	ATG / TAA	ATG / TAA	ATG / TAA
ATP6	ATG / TAA	ATG / TAA	ATG / TAA	ATG / TAA	ATG / TAA	ATG / TAA	ATG / TAA	ATG / TAA	ATG / TAA	ATG / TAA	ATG / TAA	ATG / TAA	ATG / TAA	ATG / TAA	ATG / TAA	ATG / TAA	ATG / TAA	ATG / TAA	ATG / TAA
COX3	ATG / T—	ATG / T—	ATG / TAA	ATG / T—	ATG / T—	ATG / T—	ATG / T—	ATG / T—	ATG / T—	ATG / T—	ATG / T—	ATG / T—	ATG / T—	ATG / T—	ATG / T—	ATG / T—	ATG / T—	ATG / T—	ATG / T—
ND3	ATT / T—	ATT / T—	ATT / TAA	ATT / T—	ATT / T—	ATT / T—	ATC / T—	ATC / T—	ATT / T—	ATT / T—	ATT / T—	ATC / T—	ATC / T—	ATC / T—	ATT / T—	ATT / T—	ATT / T—	ATT / T—	ATT / T—
ND4L	ATG / TAA	ATG / TAA	ATG / TAA	ATG / TAA	ATG / TAA	ATG / TAA	ATG / TAA	ATG / TAA	ATG / TAA	ATG / TAA	ATG / TAA	ATG / TAA	ATG / TAA	ATG / TAA	ATG / TAA	ATG / TAA	ATG / TAA	ATG / TAA	ATG / TAA
ND4	ATG / T—	ATG / T—	ATG / TAA	ATG / T—	ATG / T—	ATG / T—	ATG / T—	ATG / T—	ATG / T—	ATG / T—	ATG / T—	ATG / T—	ATG / T—	ATG / T—	ATG / T—	ATG / T—	ATG / T—	ATG / T—	ATG / T—
ND5	ATG / TAA	ATA / TAA	ATA / TAA	ATA / TAA	ATA / TAA	ATA / TAA	ATA / TAA	ATA / TAA	ATA / TAG	ATA / TAA	ATA / TAA	ATG / TAA	ATA / TAA	ATA / TAA	ATC / TAA	ATG / TAA	ATA / TAA	ATA / TAA	ATA / TAA
ND6	ATG / AGG	ATG / AGG	ATG / AGG	ATG / AGG	ATG / AGG	ATG / AGG	ATG / AGG	ATG / AGG	ATG / AGG	ATG / AGG	ATG / AGG	ATG / AGG	ATG / AGG	ATG / AGG	ATG / AGA	ATG / AGA	ATG / AGG	ATG / AGG	ATG / AGG
CYTB	ATG / T—	ATG / T—	ATG / T—	ATG / T—	ATG / T—	ATG / T—	ATG / T—	ATG / T—	ATG / T—	ATG / T—	ATG / T—	ATG / T—	ATG / T—	ATG / T—	ATG / T—	ATG / T—	ATG / T—	ATG / T—	ATG / T—

### Further discussion

The introduced reference-assisted *de novo* assembly with multiple k-mer strategy for mitogenome assembly consists of two steps: mitochondrial genome reads selection and *de novo* way to assemble the mitogenome. The proposed pipeline for mitogenome assembly can be used for *de novo* mitogenome assembly from mitochondrial genome sequencing library as well. Moreover, it is the critical issue to determine the closeness of reference genome with the sequencing target because selected mitochondrial genome reads will totally affect the outcome. But, it is still not clear how closely related reference genome will be affected because of the complexity of mitogenomes between species.

The initial assumption of *de novo* assembly with multiple k-mer strategy on different read pools generated by using different QV threshold attempts to identify the final mitogenome with identical scaffold supports. In this study, while we used the mitochondrial genome of Indian rhesus macaque as reference genome, the largest cluster with 7 identical sequences was identified as the final complete mitogenome of Taiwanese macaque. There are 4 copies in forward strand forms and 3 copies in reverse strand forms in the CD-HIT-EST cluster. This is a good sign that the major mitochondrial genome copy could be detected by the proposed method and identical sequences support to the final complete mitogenome. We also found that the k-mer of the identical sequences ranges from 81 to 89. These results suggested that larger k-mer have capability to assemble the complete mitogenome. In summary, the proposed method provide an alternative solution for mitochondrial genome assembly with identical sequence supports.

## Conclusions

In 1976, Fooden reported a provisional classification of 19 macaque species clustered into four groups based on the structure of male external genitalia: the silenus-sylvanus group, the sinica group, the fascicularis group, and the arctoides group [15]. *M*. *cyclopis*, together with *M*. *fascicularis*, *M*. *mulatta*, and *M*. *fuscata*, were assigned to the fascicularis group. This classification based on reproductive anatomy was later supported by molecular evidence. Recently, owing to advances in PCR and sequencing technologies, researchers were able to use mitochondrial DNA fragments (e.g., control region, rRNA or tRNA genes) for the phylogenetic study of *M*. *cyclopis* [13, 16, 42, 43]. However, due to the lack of a complete mitochondrial genome, only partial mitochondrial DNA sequences were analyzed for cross-species comparison. Here, for the first time, we assembled the mitochondrial genome of *M*. *cyclopis*, making it available for whole mitochondrial genome-based phylogenetic study.

In accordance with most of the previous reports, our whole mitogenome-base phylogenetic study indicated a strong phylogenetic tie between *M*. *cyclopis* and other macaque species in the fascicularis group, especially *M*. *mulatta lasiotus*, a species living in Southern China. Similar results were also reported by Chu et al. [13] and Smith et al. [18], which showed the strongest phylogenetic connection of *M*. *cyclopis* with macaques living in Sichuan and Yunnan area, China. Studies of 835 bp mtDNA across species of the facicularis group by Smith et al. indicated a closer phylogenetic relationship of Chinese rhesus macaque to Taiwanese macaque, than to Indian rhesus macaque [18]. These data also suggested the migration of macaques from Asian continent to Taiwan during the ice age.

## Supporting Information

S1 Dataset Raw paired-end reads for assembling Taiwanese macaque mitochondrial genome. The compressed file contains paired-end reads in FASTQ format (MitoReads.PE.R1.fastq and MitoReads.PE.R2.fastq).
(TGZ)
